# Assessment of burnout and associated factors among medical educators

**DOI:** 10.12669/pjms.37.3.3078

**Published:** 2021

**Authors:** Zareena Akram, Ahsan Sethi, Aabish Mehreen Khan, Fatima Zia Zaidi

**Affiliations:** 1 Zareena Akram, Lecturer, Department of Physiology, Poonch Medical College, AJK, Pakistan; 2 Ahsan Sethi, BDS, MPH, MMED, FHEA, MAcadMEd, FDTFEd, PhD Assistant Professor and MHPE/PhD Supervisor, Institute of Health Professions Education & Research, Khyber Medical University, Hayatabad, Peshawar, Pakistan; 3 Aabish Mehreen Khan, Demonstrator, Department of Community Medicine, King Edward Medical University, Lahore, Pakistan; 4 Fatima Zia Zaidi, Department of Medical Education, UCM, The University of Lahore, Pakistan

**Keywords:** Assessment, Burnout, Stress, Medical educators, Maslach burnout inventory

## Abstract

**Objective:**

To assess burnout in medical educators and to identify factors associated with it.

**Methods:**

A sequential mixed methods research study was conducted over eight months from July 2018 until February 2019. Participants included medical educators, who are studying for or graduated with a postgraduate qualification in medical education. An online questionnaire was developed using Maslach Burnout Inventory to collect quantitative data. The findings were explored in-depth qualitatively. Descriptive and inferential statistics were calculated for the quantitative data using SPSS 20. For qualitative data, we performed thematic analysis.

**Results:**

Of total 160 medical educationists, 101 responded giving 63.1% response rate. Mean age was 41.4 years and majority 53.5% were females. Overall aggregate mean burnout level was 12.34 ± 7.36 whereas sub-domains of Maslach burnout inventory (MBI) like i) emotional exhaustion, ii) depersonalization and iii) personal accomplishment were found out to be 19.59, 10.42 and 11.21 respectively. Most respondents had moderate 71 (70.3%) emotional exhaustion and 8 (8.9%) had severe emotional exhaustion. Average level of depersonalization was suffered by 73 (72.3%) respondents and severe level was observed in 20 (19.8%) respondents. Personal accomplishment was found low in all 101 (100.0%) respondents. Selective in-depth interviews revealed that coping mechanisms like social gatherings, indoor and outdoor game facilities and outings and leisure time should be strategized for faculties.

**Conclusion:**

In this study medical educators were found to have quite high level of burnout. The early career medical educators feels emotionally exhausted, with low sense of personal accomplishment.

## INTRODUCTION

Stress is a well-known reality in the contemporary life. Previous investigations from UK estimated that work related stress loses 20 million days each year, thus, decreasing productivity.^1^ Burnout is a syndrome which puts a worker experiencing emotional fatigue, depersonalization, reduced sense of job gratification. Due to burnout, many physical stressors arise, including gastrointestinal problems, nervousness, insomnia, and fluctuating eating patterns.^2^ The increased focus on burnout can be attributed to the increased level of knowledge regarding burnout condition and undesirable impact on people’s work.^3^ Work related stress and burnout affects all jobs and professions irrespective of white and/or blue color statuses, however medical educators face a distinctive level of stress at job.^4^

Medical educationists are under consistent pressure such as assessments, curriculum modulations, demands of students and institution, accreditation and regulatory formalities and balance in teaching and clinical performance.^5^ Most of burnout is related to financial hazards for the institution and the society in terms of long time-offs, low inspiration for work, mental sicknesses, heart problems and hospitalization. Moreover, it affects job presentation such like motivation, work excellence and accountability come true.^6^

Phenomena of good teacher is not enough now for a medical education leader as with advancement new techniques and approaches for solving issues can be inculcated by Health Professional Education (HPE) programs which uses integrated and holistic methods.^7^ Research has revealed that work-related anxiety and burnout are associated with high level of expectations and demands placed on medical educationists.^8^ When bearing in mind educators as frontline staff ready to solve a problem in crises, one must also reflect the impact of the crisis on the individual workers. 9

Though burnout status of clinicians has been documented, but very few have focused on Medical Educators in local as well as international context.^9^ It is understood that medical educators are bearing different stressors and tensions than that of clinicians, e. g. they are fronting complications while managing their multiple intersecting identities, medical educators are not appreciated and are also underpaid. These burdens could put a worker in a state of detachment, pessimism and a weak sense of identity.^10^ This study aimed to assess the burden of burnout among medical educators and to define which personal and job-related factors are associated.

## METHODS

This study was based on mix methods utilizing both quantitative and qualitative research techniques. For quantitative part, a cross sectional survey was conducted. Participants and graduates of MME/MHPE programmes from University of Lahore (UOL), Khyber Medical University (KMU), Peshawar and RIPHAH International University, Rawalpindi were study subjects. Duration was eight months from July 2018 to February 2019.

Medical Educators were defined as any person who is studying or has graduated with a postgraduate qualification such as Masters of Health Professional Education or Masters in Medical Education. In this study for quantitative analysis the interpretation of burnout was done according to its sub-domains:

Total aggregate burnout = Emotional Exhaustion + Depersonalization - Personal Accomplishment.

As regards individual components of burnout:

- Emotional Exhaustion was stratified as mild (<16), moderate (16-27) and severe (>27).
- Depersonalization as mild (<6), average (7-12) and severe (>12).
- Personal accomplishment was stratified as high (>39), average (38-32) and low (<32).

For qualitative part, a group of respondents from the quantitative survey were selected for in-depth interview on telephone. Open-ended questions were asked to assess perceptions and understanding about burnout and their coping techniques.

### Questionnaire:

Burnout was assessed by using Modified Maslach burnout inventory (MBI) - educators survey which is a validated tool for burnout assessment. A semi- structured interview consisting of open- and closed-ended questions regarding Medical Educators’ perspectives was used.^11^

### Data collection:

The Ethical Committee of University of Lahore (UOL) approved the study (letter no. ERC/03/18/12, dated 20-12-2018). A questionnaire was sent to 160 medical educators via email (response rate = 63.1%). The list of Medical Educators of MME/MHPE Programmes in the three universities was retrieved using convenient sampling. Those undergoing any known medical or psychological condition or who did not consent and respond got automatically excluded. Firstly, the baseline information in terms of age, sex, education, qualification and then information pertaining to burnout was gathered. The selection and observation bias was addressed during analysis by stratification. For qualitative part, in-depth interview of five participants was conducted.

### Data analysis:

The categorical variables like sex, education, and academic position were analyzed as frequency and percentages. Mean and standard deviations were quantified from continuous numerical variables like age, individual and aggregate MBI, and sub-domains of MBI. ANOVA was used to compare mean MBI sub-domains and aggregate according to academic positions. Thematic analysis was performed on the qualitative data. After transcription of interviews, Open coding was performed. Similar codes were merged to form sub-themes and themes. These themes were refined throughout to fit the data.

## RESULTS

A total of 101 medical educators were enrolled, 47 (46.5%) were males and 54 (53.5%) females. The mean age of respondents was 41.4 years, with majority 80.0% between 31 to 50 years. Most of the respondents had postgraduate level education i.e. FCPS/MCPS/MME or were currently taking this education. Majority were students 69 (68.3%), few were demonstrators 16 (15.7%) whereas 50 (49.5%) were Assistant Professors, and 22 (21.6%) were Professors (Table-I).

**Table I:** Baseline characteristics of participants (n=101).

	Frequency	Percentage
***Sex***		
Male	47	46.5%
Female	54	53.5%
***Age (years)***		
Up to 30	4	4.0%
31 to 40	48	47.5%
41 to 50	35	34.5%
51 or above	14	13.5%
***Education level***		
MBBS	9	8.5%
FCPS/MCPS	87	86.6%
PhD	5	4.8%
***Academic status***		
Student	69	68.3%
Graduate	32	31.6%
***Academic position***		
Demonstrator	16	15.7%
Assistant Professor	50	49.5%
Associate Professor	13	12.7%
Professor	22	21.6%

Most respondents had moderate 71 (70.3%) level emotional exhaustion whereas 9 (8.9%) had severe level. Average level of depersonalization was seen in 73 (72.3%) and severe level was observed in 20 (19.8%) respondents. Personal accomplishment was found low in all 101 (100.0%) respondents. The overall aggregate mean burnout level was 12.34 ± 7.36 showing high burnout while sub-domains i.e. emotional exhaustion, depersonalization and personal accomplishment were found to be 19.59, 10.42 and 11.21 respectively showing burnout in all respondents (Table-II).

**Table II:** Level of burnout in the study (n=101).

	Frequency	Percentage
***Emotional exhaustion***		
Mild	21	20.8%
Moderate	71	70.3%
Severe	9	8.9%
Mean ± SD	19.59 ± 5.49	
***Depersonalization***		
Mild	8	7.9%
Average	73	72.3%
Severe	20	19.8%
Mean ± SD	10.42 ± 2.77	
***Personal accomplishment***		
Low	101	100.0%
Average	0	0.0%
High	0	0.0%
Mean ± SD	18.71 ± 2.30	
Overall aggregate burnout*		
Mean ± SD	12.34 ± 7.36	

* Aggregate burnout = Emotional exhaustion + Depersonalization - Personal accomplishment.

When different demographic characteristics were compared according to academic positions in terms of average sub-domains of emotional exhaustion, depersonalization and personal accomplishments it was noted that male demonstrators were emotionally more exhausted (23.75 versus 20.41 and 15.60 respectively). Similarly, demonstrators from Riphah International University experienced more emotional exhaustion (p-value, 0.03). The full-time demonstrators were also significantly emotionally exhausted than professors (23.23 versus 9.75). Furthermore, male demonstrators were experiencing more depersonalization than senior faculty (12.38 versus 8.80, p-value 0.02). The demonstrators from KMU had significantly high depersonalization than Assistant/Associate Professors and Professors (13.67 versus 11.27 and 7.25 respectively). Full-time demonstrators had high level of depersonalization (12.38 versus 6.75). Personal accomplishment was found significantly high in Professors from KMU irrespective of their working full time or part time (p-value, 0.003). The level of personal accomplishment was also high in female Professors, however, not proven significant (Table-III).

**Table III:** Comparison of mean burnout subscales according to demographic profile between academic positions.

		Demonstrators (n=16)	Assistant/Associate Professors(n=63)	Professors (n=22)	p-value
	Male	23.75 (4.1)	20.41 (4.34)	15.60 (5.73)	0.002*
	Female	20.75 (8.20)	19.85 (4.90)	16.67 (5.82)	0.19
	UOL	21.83 (7.16)	19.90 (4.41)	18.29 (6.02)	0.48
Emotional Exhaustion	RIU	21.86 (7.51)	17.36 (3.41)	14.73 (5.19)	0.03*
	KMU	24.05 (3.01)	21.77 (4.62)	16.50 (6.65)	0.09
	Full time	23.23 (6.77)	18.80 (4.22)	9.75 (2.06)	<0.001*
	Part time	18.04 (2.00)	20.52 (4.71)	17.61 (5.20)	0.08
	Male	12.38 (3.29)	10.59 (2.26)	8.80 (2.86)	0.02*
	Female	11.75 (2.76)	10.41 (2.47)	9.17 (3.43)	0.12
	UOL	12.17 (2.78)	10.23 (2.31)	9.43 (3.20)	0.14
Depersonalization	RIU	11.29 (3.03)	9.64 (1.85)	9.36 (3.35)	0.34
	KMU	13.67 (3.51)	11.27 (2.51)	7.25 (2.21)	0.007*
	Full time	12.38 (3.17)	10.00 (2.75)	6.75 (3.50)	0.007*
	Part time	10.67 (1.15)	10.65 (2.23)	9.50 (2.89)	0.22
	Male	18.12 (0.64)	18.38 (2.45)	19.70 (1.94)	0.20
	Female	18.88 (1.12)	18.32 (2.35)	20.08 (2.72)	0.08
	UOL	18.33 (1.30)	18.13 (2.19)	19.43 (1.27)	0.30
Personal Accomplishment	RIU	18.86 (1.06)	19.82 (2.71)	19.91 (3.20)	0.68
	KMU	18.01 (0.59)	17.91 (2.28)	20.75 (0.95)	0.05
	Full time	18.23 (0.83)	19.93 (1.98)	21.50 (2.38)	0.003*
	Part time	19.67 (0.57)	19.85 (2.29)	19.56 (2.30)	0.01*

* Statistically significant p-values

### MBI categorization:

- Emotional Exhaustion was stratified as mild (<16), moderate (16-27) and severe (>27).
- Depersonalization as mild (<6), average (7-12) and severe (>13).
- Personal accomplishment was stratified as high (>39), average (38-32) and low (<31).

In-depth interviews of respondents revealed identity and survival issues as other departments and faculty don’t give weightage to guidelines and protocols developed by medical education departments. One respondent claimed that *“It’s a headache when we try to explain each and every component of medical education”*. Another said that *“my work as medical educationist has no identity, no recognition of department by faculty*.

Poor job structure and absence of formal mentoring are additional factors, other departments do not understand role of medical education. It leads to frustration, when people don’t accept change especially, as medical educationists develop and revise the curriculum standards etc. and when they are not supported or taken seriously, all goes to waste.

Key coping strategies were reading, family outing, physical exercise and walking. One participant said that *“Family and friends help in these situations. I plan meditation, reading and exercise and walk. Family outing etc.”*

Most of the educationists do not know whether they are having burnout or not. *One interviewee said* “I have the support of my colleagues. My peers are my only support in the whole system. When stressed I also think of my family, my children and spouse”.

Some of the respondents suggested solutions for working environment of medical educators. As most of them were apparently victims of current working environment.

### Interviewee one:

“I would have a way of removing political influences completely and have a fair world, whereby people get job based on competence and the right person for the right job”.

### Interviewee two:

“I would communicate to the higher management, to give firm authority to medical education department in making certain important decisions and recommendations”.

## DISCUSSION

The current study highlights a very significant level of burnout in medical faculty working in different medical colleges and universities across Pakistan. Overall medium level of burnout was noted in 88.0% cases whereas remaining 12.0% had high level of burnout. Several previous studies have highlighted burnout in healthcare workers specially in physicians. A previous study from Karachi witnessed 10% respondents with severe level burnout.^12^ Another military hospital based study also revealed high level of severe burnout (27.8%) in doctors.^13^ The current study found moderate or medium level burnout. A study from Egypt noticed high frequency of moderate burnout in faculty.^14^ Burnout is a psychological condition where the level of work related stress is high due to which motivation and dedication of workers becomes low.

In the present study younger age had higher level of burnout, similarly, those working on Demonstrators and Assistant Professors positions were suffering from moderate to severe level of emotional exhaustion and depersonalization. A local study by Mazhar SB et al reported that 4^th^ year postgraduate residents had high level of burnout. They also witnessed that severe burnout was (23.4%) and majority of faculty had moderate burnout (44.7%).^15^ Current study validates many previous evidence base from local as well as international settings.

In the current study significantly high mean levels of emotional exhaustion, depersonalization and low level of personal accomplishments was noticed between demonstrators, Assistant Professor and senior faculty of Associate Professors and Professors. Similarly, younger age and graduate level education (MBBS) had comparatively higher burnout. A Turkish study by Armutlukuyu M et al noticed that mean emotional exhaustion (EE) and depersonalization (DP) (p<0.001) scores were significant in physicians working in basic sciences, the staff titled Professor, Associate Professor, the staff at/over 40 years of age and married physicians than those of the staff employed in the other medical and surgical sciences departments, the staff with other titles, under 40 years of age and those whose marital status was single.^16^ Tijdink JK and colleagues also noticed that younger academic professionals had higher mean emotional exhaustion.^17^ Yu J et al. from Korea reported that significant differences were present in the level of burnout across different faculty position levels.^9^ This huge evidence based on burnout is a proof that medical academicians are more prone to burnout. Since faculty members working as teachers as well as clinicians are consistently busy, this group is on verge of psychological exhaustion due to high burden.

The association of burnout with younger ages and early professional career is to some extent understandable. In job sectors of developing countries like Pakistan the future is uncertain and this keeps the early entrants on toes. The medical training at undergraduate and postgraduate levels are very competitive and tough and if this routine continues in professional career, it becomes a chronic stress. This burned out academician’s situation is very challenging for healthcare programmers and administrators. The coping strategies and counseling plays an important role. Since their output affects patients and medical students alike, various interventions to reduce burnout, specially faculty teaching undergraduate and postgraduate students are present.

During in-depth interviews, most of the interviewees mentioned that they have identity issue as others don’t recognize importance of medical education department. Other key factors faced by were poor job structure and absence of formal mentoring. Interviewees also confessed that their stress or burnout is unknown so they suggested periodic examinations and counselling.

There were multiple advantages and key findings of this study; firstly, there were very few previous studies targeting burnout in medical educators, and this one of the initial evidence showing significant burnout in educators. Secondly, a representative sample from both public and private medical institutions was selected, thus giving scenario of both healthcare setups. And both quantitative and qualitative assessments were done revealing great insight into the topic.

### Limitations of the study:

It was email based survey and many participants were sent multiple reminders still one third did not respond. Due to lack of time some interviewees could not respond in detail and merely replied qualitatively (yes, no) so others were approached to fill their place and complete five interviews.

## CONCLUSION

The current study has found high level of burnout in medical educators, almost all study respondents were suffering from moderate to medium level of burnout in this study. Emotional exhaustion and depersonalization were of moderate level high, whereas personal accomplishment was low in all respondents.

### Suggestions:

Medical institutions should provide a calm and workable environment to its staff. Coping mechanisms should be strategized for faculties specially the younger professionals. Further research is required to assess various interventions for the management of job related stress and burnout.

### Authors’ Contributions:

**ZA and AS** conceptualized and planned the study.

**ZA** collected data and conducted analysis and wrote first draft of manuscript.

**AM and FZZ** helped in analysis and writing of manuscript.

**AS** conducted critical review of the draft manuscript.

**FZZ** revised the manuscript.

**ZA** on behalf of all authors will be responsible for authenticity of data and integrity of write-up.
